# Novel Vaginoplasty Technique for Management of Vaginal Agenesis and Uterine Cervix Atresia: A Case Report

**DOI:** 10.7759/cureus.102987

**Published:** 2026-02-04

**Authors:** Shumpei Goto, Taro Ikeda, Hideo Nakai, Eri Nagasaki, Kenro Chikazawa

**Affiliations:** 1 Department of Pediatric Surgery, Jichi Medical University Saitama Medical Center, Saitama, JPN; 2 Department of Urology, Jichi Medical University Saitama Medical Center, Saitama, JPN; 3 Department of Gynecology, Jichi Medical University Saitama Medical Center, Saitama, JPN

**Keywords:** case report, cervical defect, pediatrics, vaginal agenesis, vaginoplasty, yang-monti tube

## Abstract

Various methods for vaginoplasty exist, but the choice depends on anatomy and body size. Conventional vaginoplasty, when performed with bowel segments rather than prosthetic materials, allows modification of canal length but lacks precise control of diameter, rendering it less suitable for pediatric patients and for those with small pelvic cavities. Moreover, the positional traction sometimes necessary under these conditions may compromise mesenteric perfusion and heighten the risk of ischemic complications. This report introduces a novel technique using the Yang-Monti tube principle with end-to-end anastomosis. A 12-year-old girl with type II Mayer-Rokitansky-Küster-Haüser syndrome (MRKH), left hydronephrosis, and hematometra was admitted with abdominal pain. Emergency drainage resolved her symptoms, and imaging revealed a unicornuate uterus, cervical atresia, and vaginal agenesis. A 7 cm sigmoid colon segment was used to create a vaginal canal, connecting the cervix and vaginal vestibular mucosa using the Yang-Monti method. The postoperative course was uneventful; the stent drain was removed after two weeks, and she was discharged on the 23rd day after learning self-care with a Nelaton catheter. Four years post-surgery, she remains healthy, with no complications such as infection or dysmenorrhea. This approach is a useful option for vaginal agenesis with cervical atresia in adolescents with a small pelvis.

## Introduction

Uterine cervical atresia is a rare congenital anomaly caused by developmental issues in the Müllerian ducts, often accompanied by vaginal malformations. Its exact incidence remains unknown, and no standardized surgical treatment has been established despite various reported methods [1]. Various surgical options for vaginoplasty have been reported. In addition to non-invasive techniques such as the Frank method [2], which achieves vaginal formation by applying pressure to the vaginal vestibule using dilators, other approaches include: methods that create a neovagina and employ substances or materials to promote epithelialization or graft take, such as split- or full-thickness skin grafts, pedicled grafts, amniotic membrane, peritoneum, oral mucosa, Interceed, or artificial dermis [3,4]; techniques utilizing intestinal segments as the vaginal canal, including small intestine, sigmoid colon, or ileocecal segment [5,6]; and procedures that elevate the vaginal vestibule using balloons or dedicated devices to construct the neovagina [7,8]. We present a case of uterine cervical atresia and vaginal agenesis in an adolescent, successfully treated with vaginal reconstruction and uterine anastomosis using the Yang-Monti (YM) technique. Our method is considered to be extremely useful in children because it utilizes the intestinal canal, thus reducing the need for repeated postoperative bougie. The YM tube allows adjustment of the thickness and is therefore easily accommodated in the narrow pelvic cavity. The relatively sufficient length of the vascular stalk may reduce the risk of blood flow disturbance due to tension caused by traction on the mesentery. Although the dilation may be necessary for intercourse in the future, we believe that the risk of cervical closure is low owing to the use of the intestinal tract. This report adheres to the CARE guidelines [9].

## Case presentation

A 12-year-old girl presented with lower abdominal pain. Her medical history included congenital esophageal atresia, status post-surgical repair at four months of age; valvular pulmonary artery stenosis under ongoing observation; acetabular dysplasia; a hypoplastic right kidney; sensorineural hearing loss managed with hearing aids; a spinal filum lipoma status post-surgical resection at four years of age; and lower-limb hypoplasia treated with growth-inhibition therapy. There is no family history, and no genetic etiology has been identified. Diagnosed with type II Mayer-Rokitansky-Küster-Haüser syndrome (MRKHS) due to a uterine hematoma accompanied by unilateral renal agenesis and vertebral abnormalities, she experienced recurrent abdominal pain despite analgesics. Imaging revealed hematometra and left hydronephrosis. On admission, she was 141 cm tall (third percentile), weighed 32.6 kg (third percentile), and had sexual characteristics consistent with Tanner stage III. Examination showed abdominal distension and tenderness. Blood and urine tests were normal.

**Figure 1:**
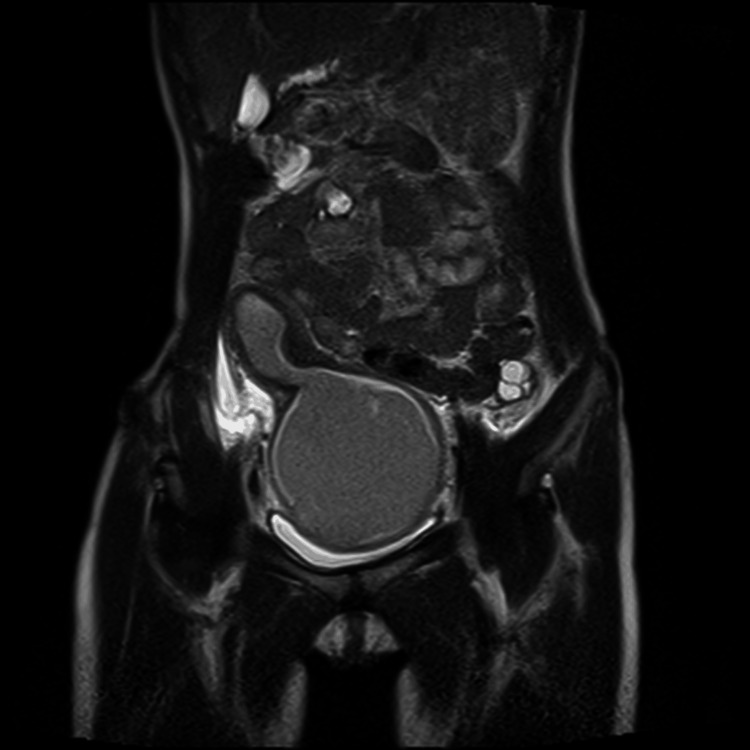
Predrainage MRI findings. On magnetic resonance imaging, a malformed uterus with intraluminal blood accumulation was observed. The enlarged uterus appears to be a contiguous adnexal horn originating from the main body of the uterus.

Ultrasound revealed a thick-walled, muscular cyst above the bladder. MRI (Figure 1) confirmed a deformed uterus with blood retention, vaginal aplasia, and hydronephrosis caused by hematometra compressing the bladder and ureter. Emergency laparotomy drained the hematometra, resolving her pain and hydronephrosis. Postoperative imaging and surgical findings confirmed a unicornuate uterus with cervical atresia and vaginal agenesis.

**Figure 2:**
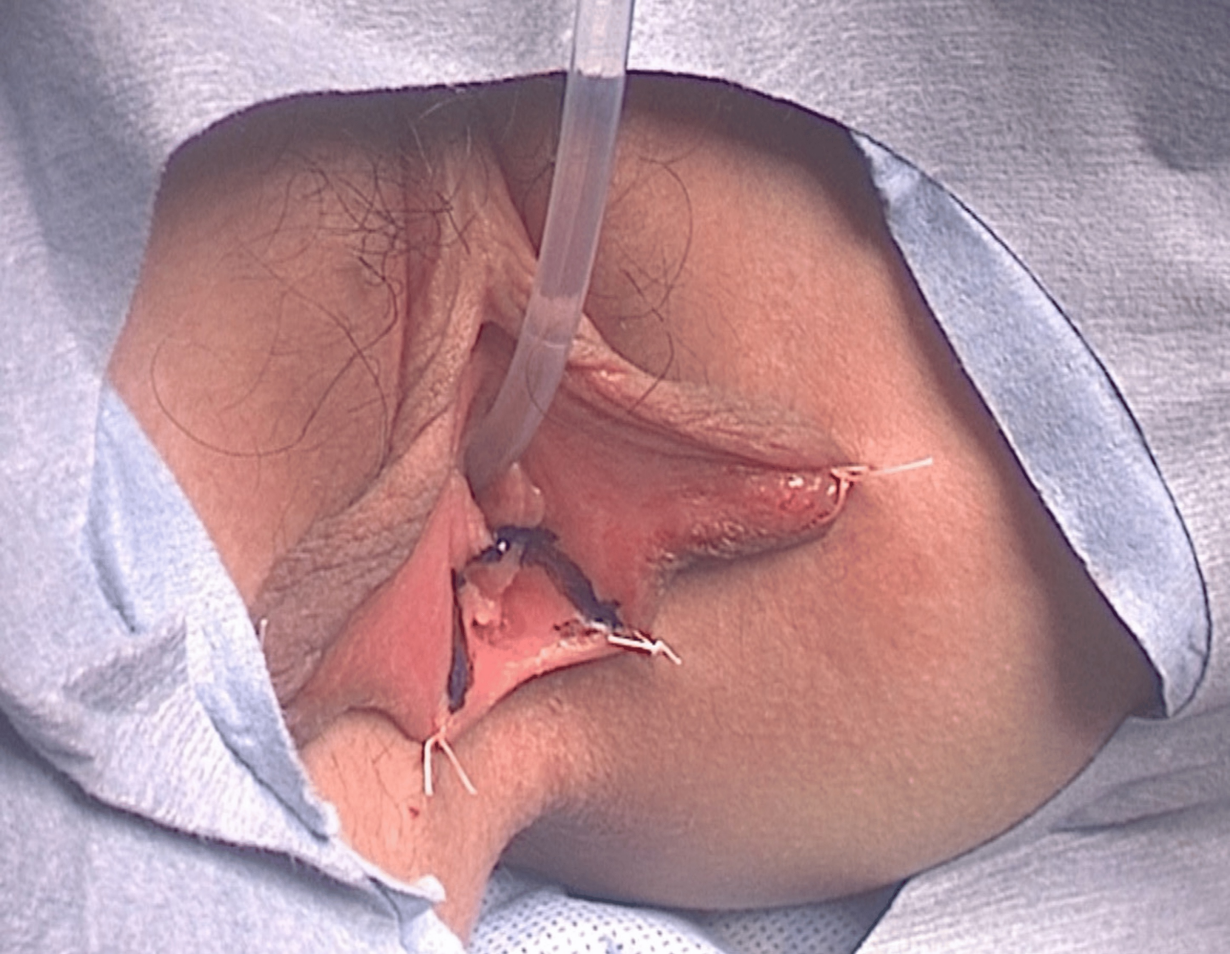
An inverted U-shaped incision in the vaginal vestibule.

Considering the distance between the uterus and perineum, pelvic cavity size, and risk of postoperative stenosis, we chose a novel approach. A 7 cm sigmoid colon segment was harvested using the YM technique to create a YM tube, which was anastomosed to the cervix and vaginal vestibule. Assuming that a tube length of 9 cm was required, and that a 7 cm segment of bowel with a diameter of 3 cm was available, we calculated that it would be possible to construct a YM tube measuring approximately 9 cm in length with an estimated diameter of about 2 cm. Given our concern regarding the length of the mesentery, which was a key issue in performing this procedure, we selected the sigmoid colon as the segment most likely to provide sufficient vascular pedicle length. Thirty-nine days after drainage, vaginal reconstruction was performed via an abdominoperineal approach. From the perineal side, an inverted U-shaped incision (Figure 2) was made to create a space for the YM tube, while the cervix was exposed via a lower abdominal incision.

**Figure 3:**
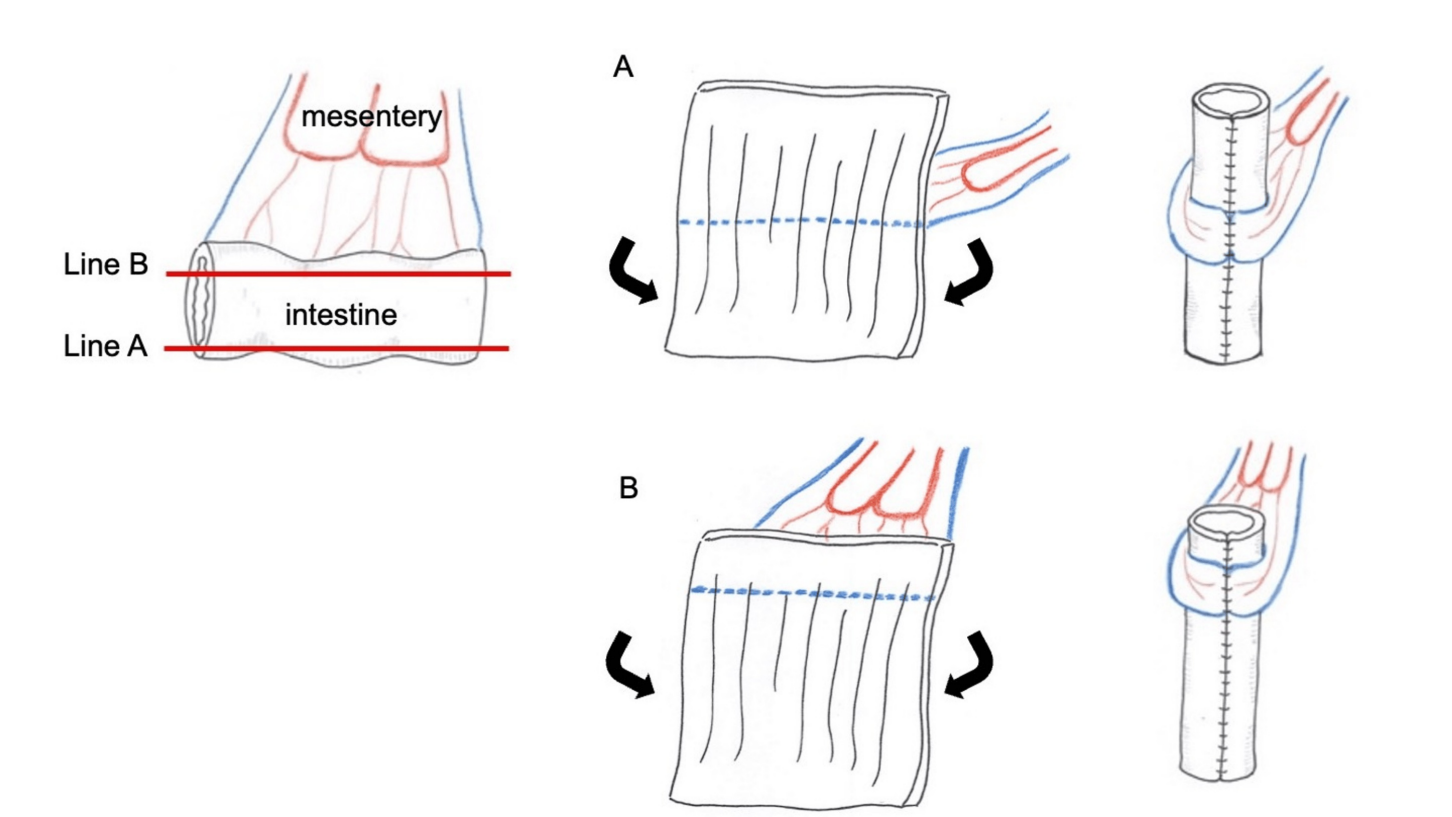
Technique for constructing the Yang-Monti tube. Incision along line A represents the conventional approach in which the incision is made opposite the mesenteric attachment, resulting in a bilaterally distributed vessel-free area above and below the mesentery. Incision along line B illustrates the modified technique used in this case, where the incision is placed closer to the mesentery with a unilateral vessel-free region. This figure was created by the authors using Preview (Apple Inc., Cupertino, CA).

**Figure 4:**
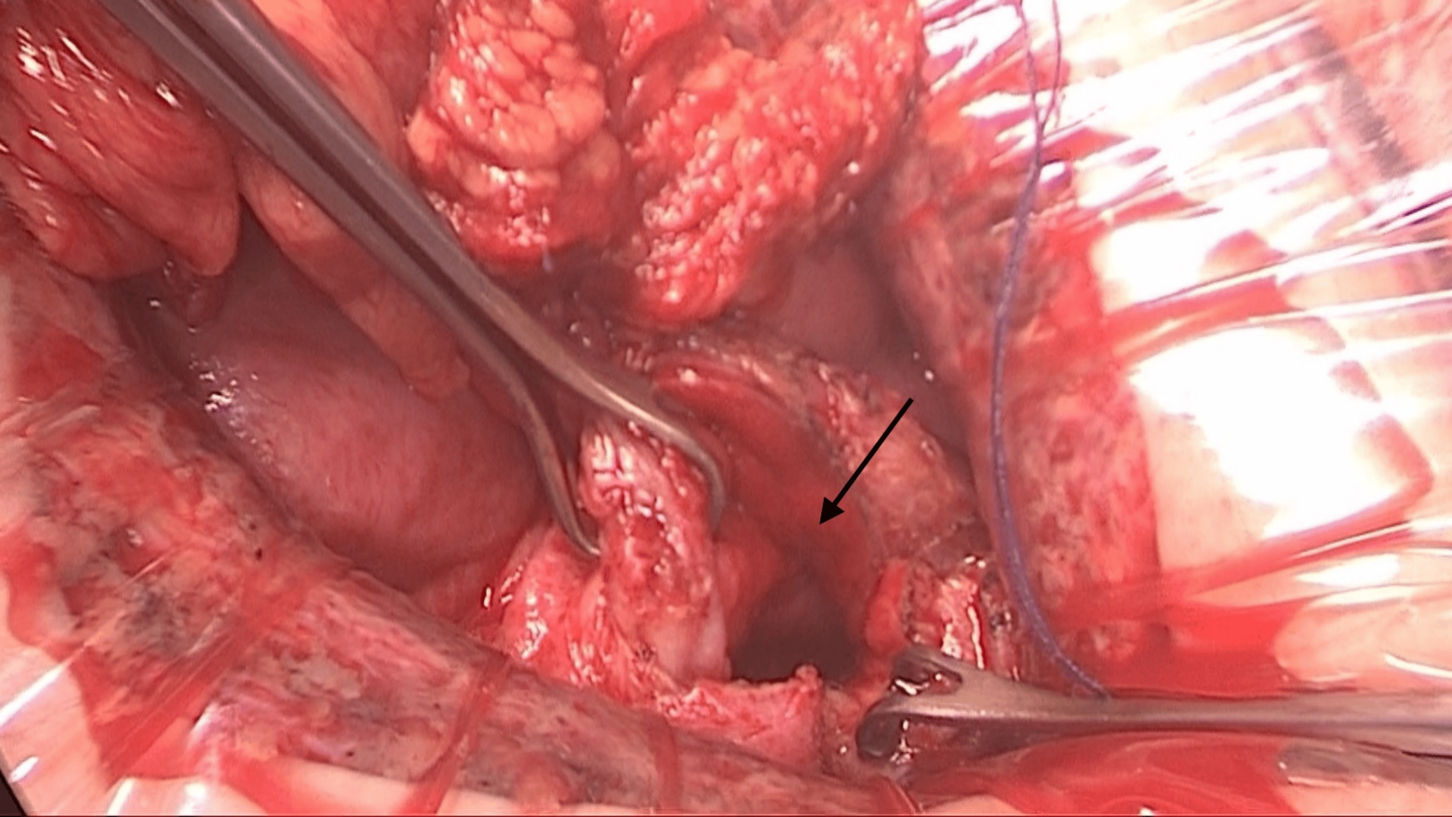
Site of uterine anastomosis. A wide incision was created at the most caudal portion of the uterine wall to facilitate anastomosis. The arrow indicates the incision in the uterine wall.

A vascularized 7 cm sigmoid colon segment was prepared to create the YM tube, ensuring the vessel-free region extended to the vestibule for optimal anastomosis (Figure 3). The YM tube was positioned in the created space, with its caudal end anastomosed to the vestibular mucosa and its cephalic end to the uterine wall (Figure 4). A 20 Fr silicone catheter was placed in the uterus as a stent drain to prevent stenosis.

**Figure 5:**
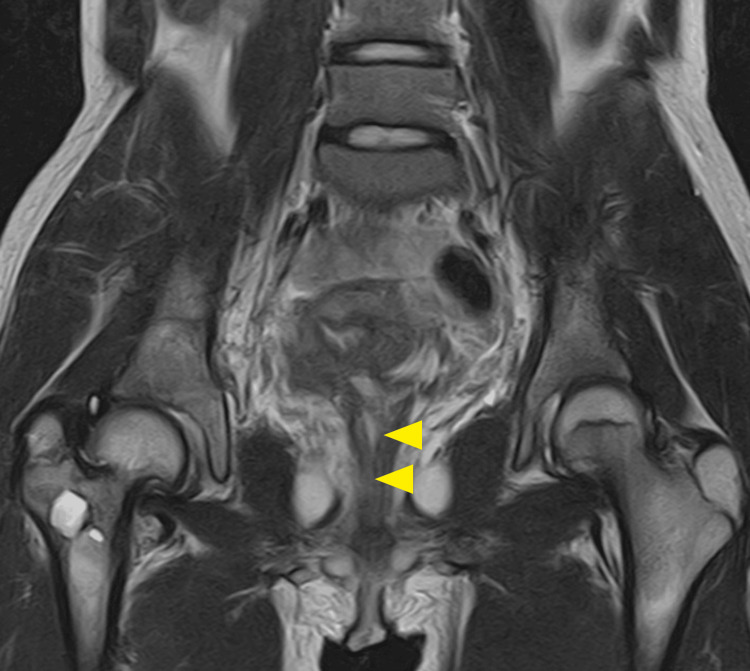
Coronal MRI image at approximately three months postoperatively. No fluid collection or effusion was identified within the reconstructed vaginal canal.

**Figure 6:**
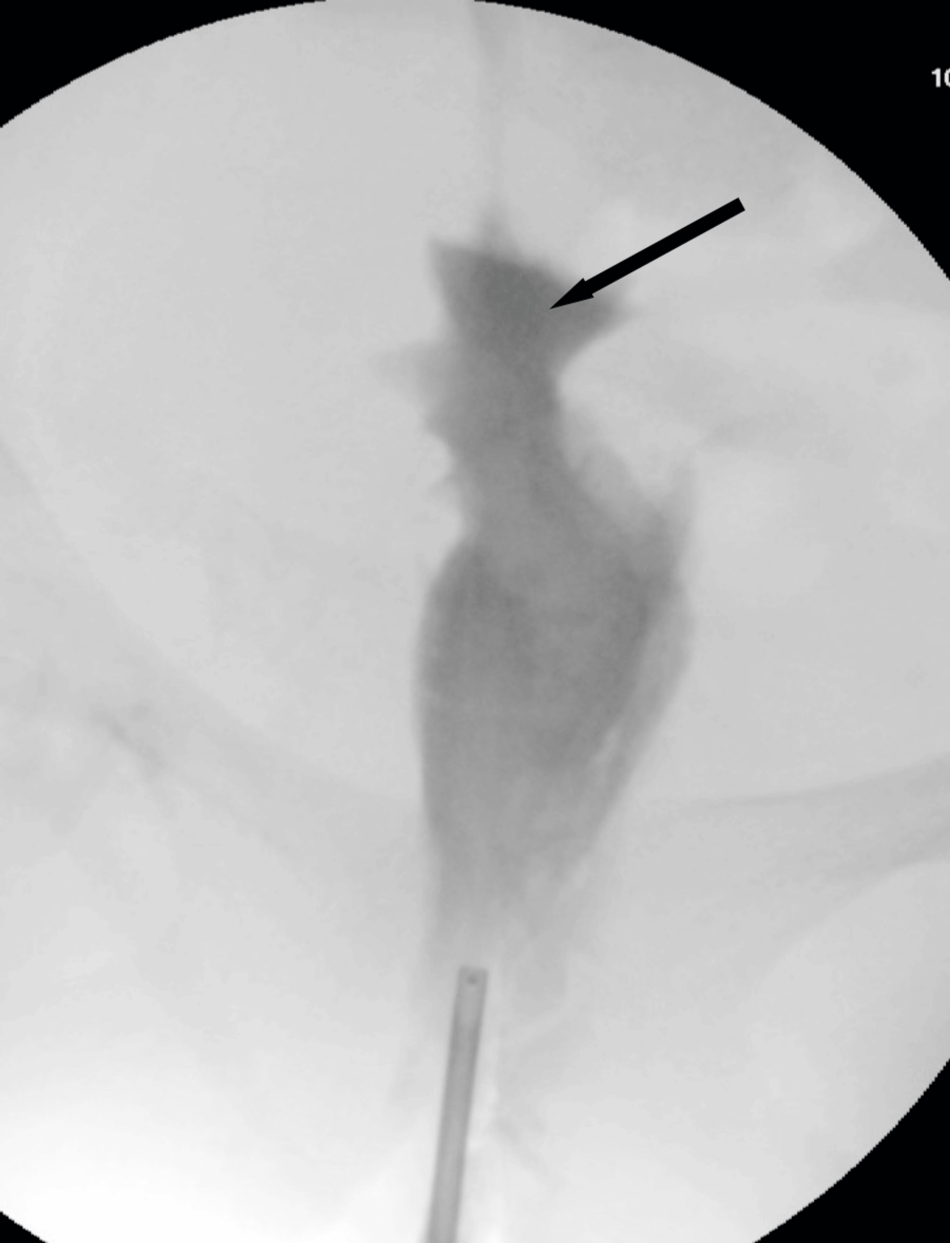
Vaginal contrast study at approximately three months postoperatively. The vaginal canal remained patent without evidence of stenosis and was clearly visualized as a lumen even under low-pressure contrast injection. The arrow indicates the site of cervical anastomosis. The uterine cavity was not visualized.

**Figure 7:**
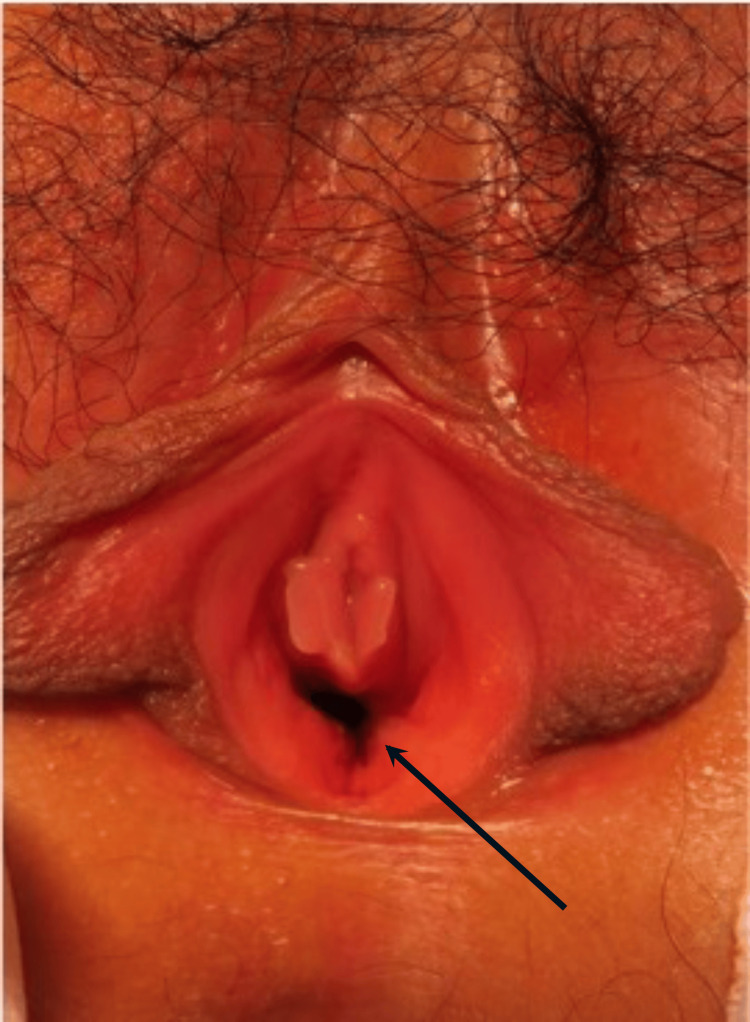
Postoperative appearance two years after vaginoplasty. The reconstructed vagina maintained an adequate and widened diameter.

The patient’s postoperative course was uneventful. The stent drain was removed two weeks after surgery, and the patient was discharged on postoperative day 23 after learning self-dilation with a Nelaton catheter. Follow-up imaging at three months, including vaginography and MRI, revealed no stenosis or abnormalities (Figures 5-7). Now, over four years post-surgery, the patient is asymptomatic, with no complications such as dysmenorrhea or infection.

## Discussion

Uterine and vaginal malformations often remain undetected before menarche due to the absence of symptoms. After puberty, conditions such as uterine aplasia or MRKHS cause primary amenorrhea, while cases of menstrual obstruction lead to hematometra, presenting with abdominal pain, masses, or urinary and defecation difficulties. Treatment is required to address these symptoms.

Cervical atresia was first described by Ludwig in 1900, with the first surgical repair reported in 1959 [10,11]. Some experts advocate for hysterectomy due to the risks of reclosure, stricture, and infection following uterine-vaginal anastomosis [12]. However, recent advances in uterine-preserving surgeries, including vaginoplasty with intestinal segments, have shown promising outcomes [13-16]. Although invasive, intestinal vaginoplasty offers durability, easier postoperative management, and superior curative potential compared to non-intestinal alternatives.

The YM technique, initially described for ureteral reconstruction, involves creating a tube from the intestine. In this case, it was adapted for vaginoplasty with uterine anastomosis. This technique has several advantages for pediatric patients. The YM tube's adjustable diameter and vascularized length are suitable for small pelvic cavities and reduce the risk of blood flow disruption. Additionally, its use minimizes the need for postoperative dilation, a significant benefit for adolescents.

The timing of surgery should be individualized. Early intervention is crucial in cases of functional uteri to prevent complications like endometriosis, reported in 56% of cervical atresia patients. Delayed surgery increases endometriosis risk, particularly when the time between symptom onset and surgery exceeds one year [17]. Hormonal therapy can be used to postpone surgery for non-menstrual indications, such as vaginal reconstruction for sexual intercourse. In this case, early surgery was chosen due to the patient’s stable physical growth and underlying conditions.

This is the first report of vaginoplasty using the YM tube with uterine anastomosis. Previous applications of the YM tube in vaginoplasty lacked a uterine connection [18].

Despite its advantages, the technique has limitations. The YM tube’s length depends on the diameter of the intestinal segment, which may not suffice for larger patients requiring longer vaginal canals. Because a partial uterine fenestration has been performed, delivery is expected to be difficult. However, it is anticipated that the patient will achieve menstruation comparable to her peers and will likely be able to engage in sexual intercourse. Another limitation is that, due to the patient’s age and our limited clinical experience in this context, it is currently difficult to provide a definitive assessment of future sexual function. Continued follow-up will therefore be necessary. Nonetheless, this approach effectively addressed the patient’s needs, and both the patient and her family expressed satisfaction with the outcome.

## Conclusions

This case highlights the potential of the YM tube technique for vaginal reconstruction with uterine anastomosis in adolescents. While the follow-up period (four years) is relatively short, the patient achieved favorable outcomes, including menstrual control and preserved uterine function. Further studies are required to evaluate long-term outcomes, particularly regarding future sexual function. However, this method represents a promising option for treating cervical atresia and vaginal agenesis in adolescents.
